# Supplementary material for: Thai Curcuma Species: Antioxidant and Bioactive Compounds
Source: Foods. 2020 Sep 2;9(9):1219. doi: 10.3390/foods9091219 (PMC7555267; doi:10.3390/foods9091219)
Supplement: Supplementary file 1 [file foods-09-01219-s001.pdf]

Table S1. List of *Curcuma* 23 species

No.	Vernacular name of *Curcuma* species	BK Herbarium No.	Deposit No.
1	*Curcuma* 'Wan Ma-Leung'	067677	MRG001
2	*Curcuma mangga* Val ex. Zijp 'Wan Khamin- Khao'	067614	MRG002
3	*Curcuma* 'Wan Ma-Hor'	067678	MRG003
4	*Curcuma* 'Wan Khamin-Dam'	067679	MRG004
5	*Curcuma* 'Wan Rang- Jud'	067680	MRG005
6	*Curcuma aeruginosa* Roxb. 'Wan Maha-Mek'	067681	MRG006
7	*Curcuma comosa* Roxb. 'Wan Chak- Mod- Luk'	067682	MRG007
8	*Curcuma* 'Wan Kanta- Mala'	067683	MRG008
9	*Curcuma aurantiaca* Van Zijp. 'Wan En-Leung'	067684	MRG009
10	*Curcuma aromatica* Salisb. 'Wan Nang-Kam'	067612	MRG010
11	*Curcuma latifolia* Rosc. 'Wan Chak- Mod- Luk'	067685	MRG011
12	*Curcuma zedoaria* Rosc. 'Wan Khamin-Oil'	067613	MRG012
13	*Curcuma longa* L.'Wan Khamin-Chan'	067686	MRG013
14	*Curcuma parviflora* 'Wan Muang'	067687	MRG014
15	*Curcuma angustifolia* Roxb. 'Wan Khai-Khun'	067688	MRG015
16	*Curcuma* 'Wan Khabitong'	067689	MRG016
17	*Curcuma* 'Wan Pataba'	067690	MRG017
18	*Curcuma* 'Wan Kortong'	067691	MRG018
19	*Curcuma* 'Wan Na-Natong'	067692	MRG019
20	*Curcuma petiolata* Roxb. 'Wan Mahachakkapad'	067693	MRG020
21	*Curcuma* 'Wan Khamin-Khao-Padtalod'	067694	MRG021
22	*Curcuma* 'Wan Chai-Dam'	067695	MRG022
23	*Curcuma* 'Wan Khamintong'	067696	MRG023

Fig S1. Spectral data for the isolated compounds.
Curcumin (1)

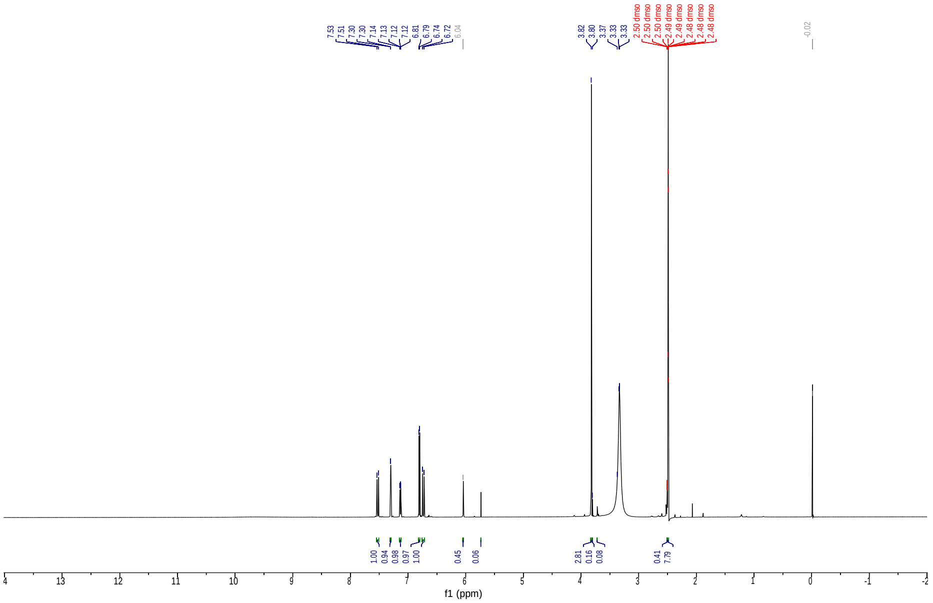

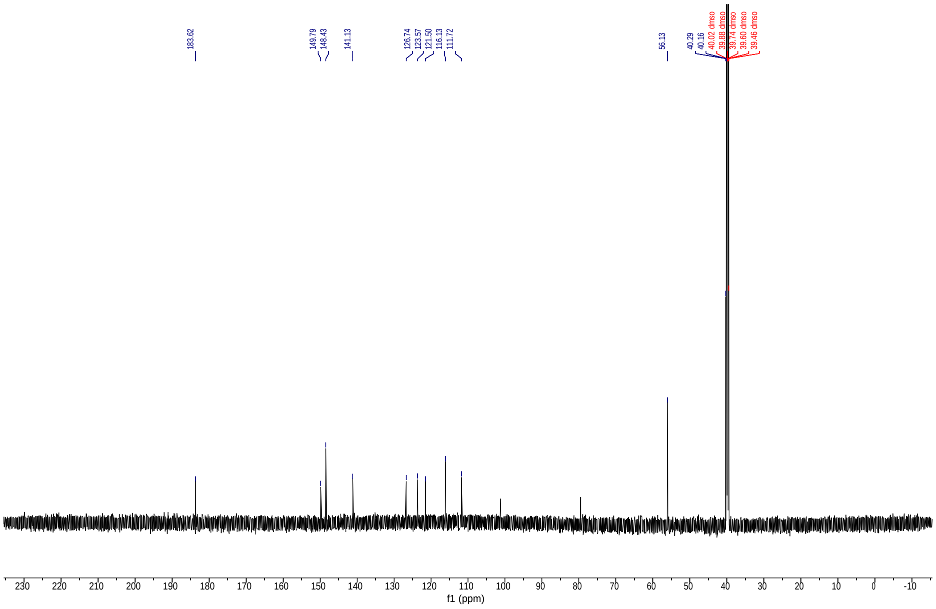

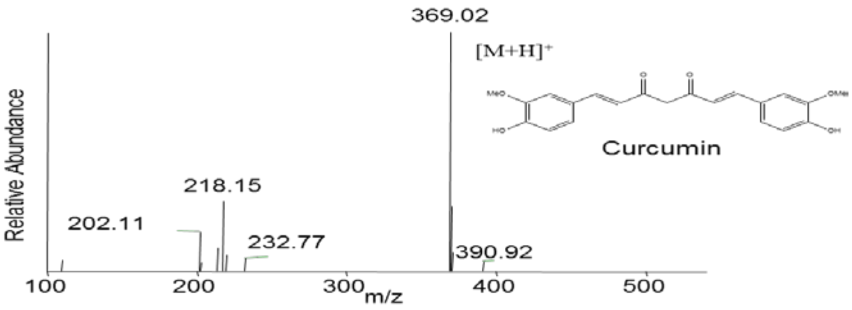

Demethoxycurcumin (2)

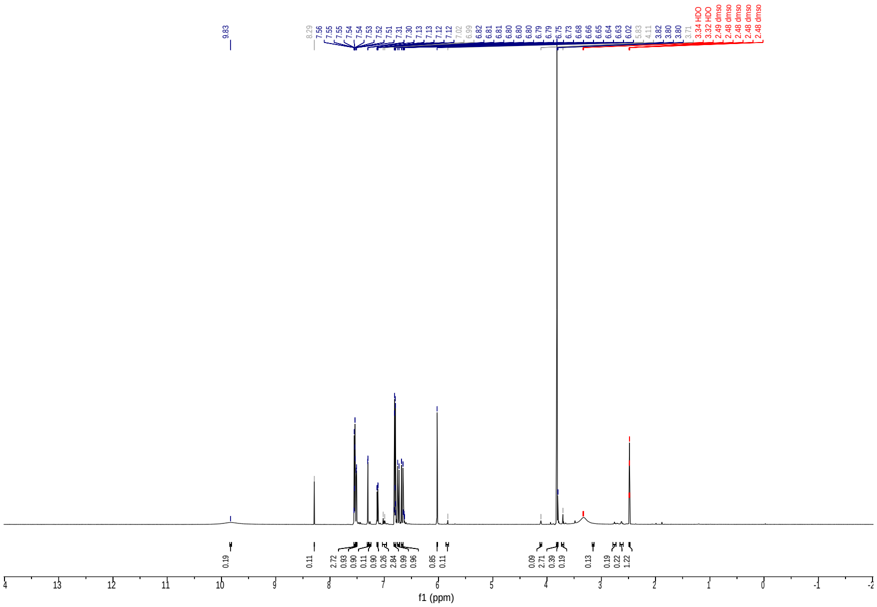

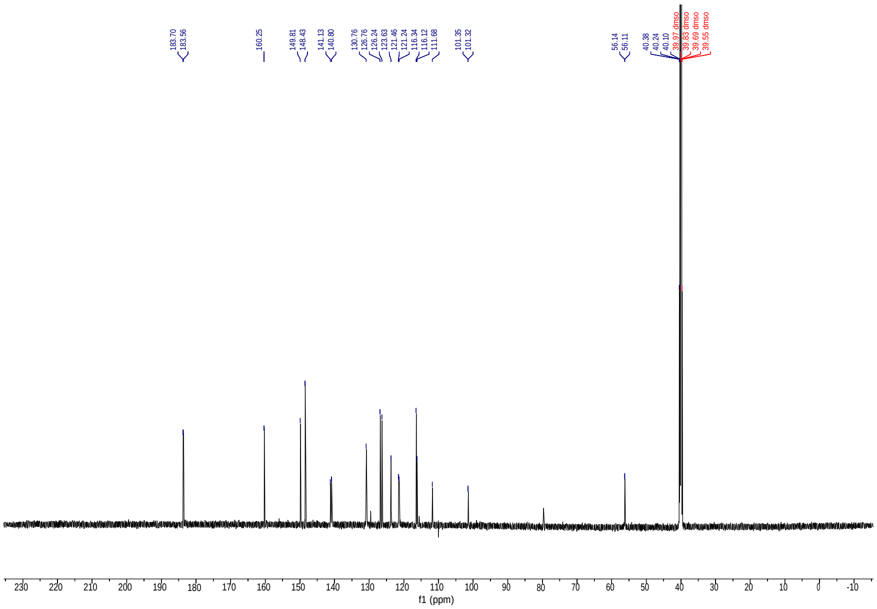

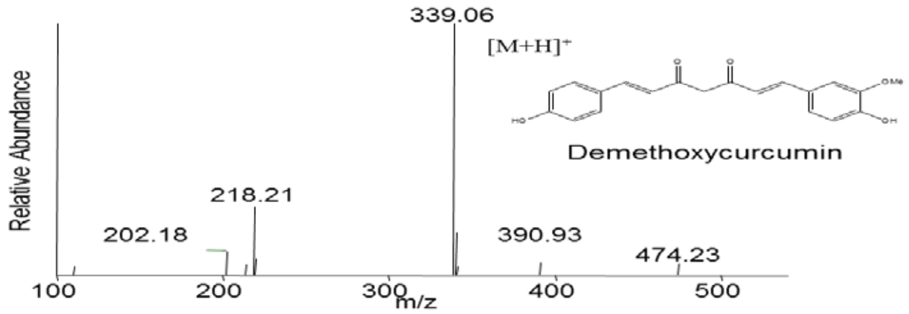

Bisdemethoxycurcumin (3)

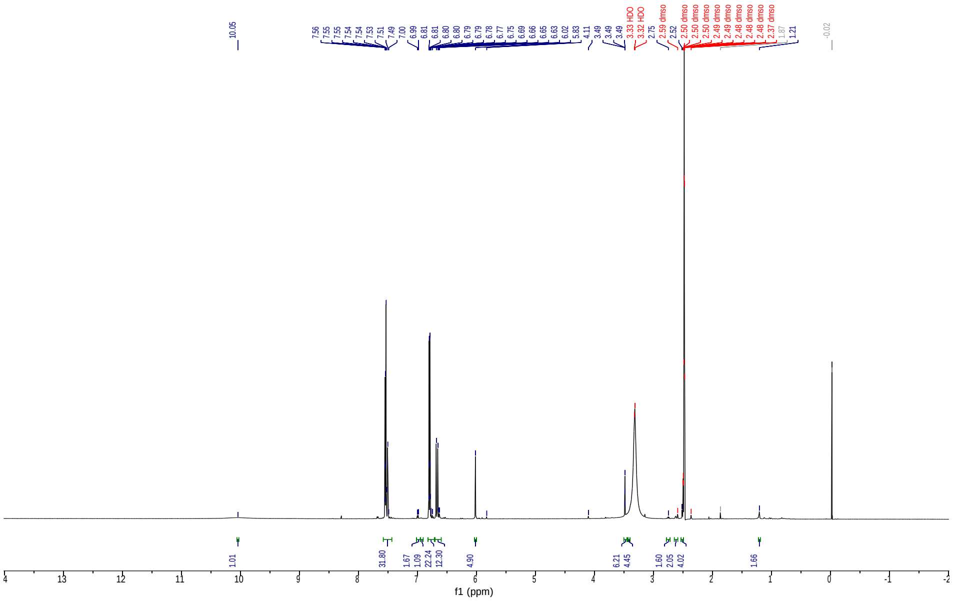

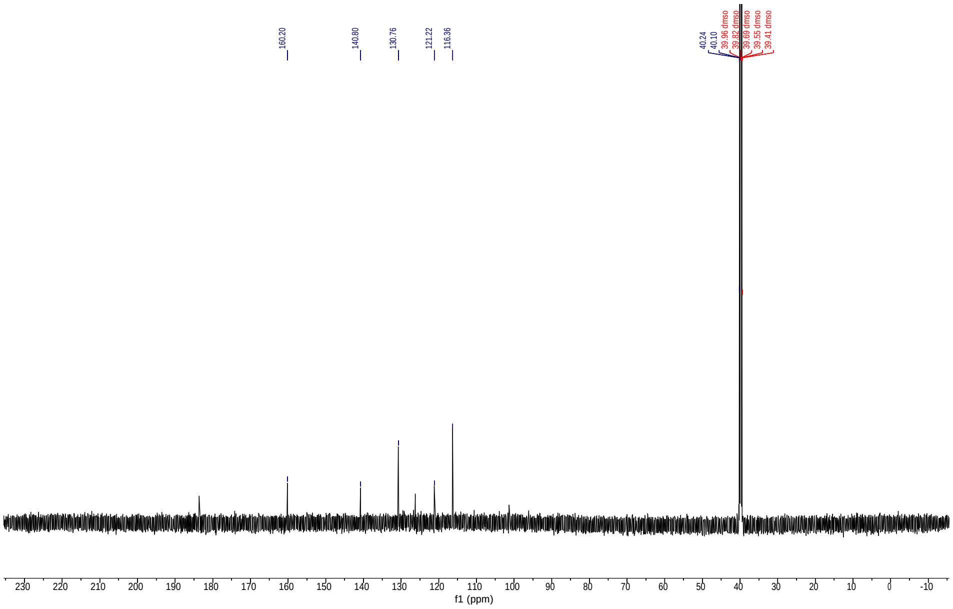

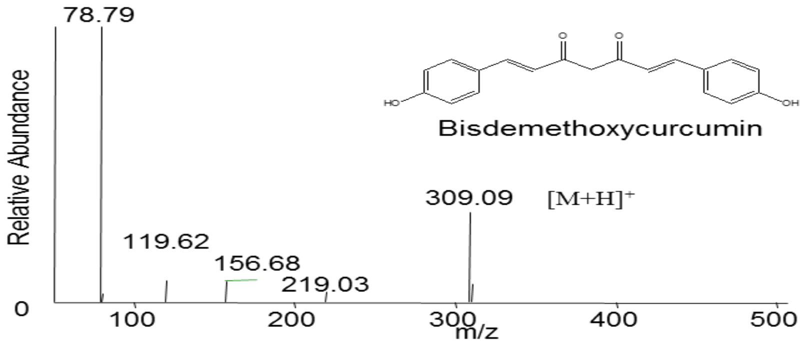

1,7-diphenyl-(4*E*,6*E*)-4,6-heptadien-3-ol (**4**)

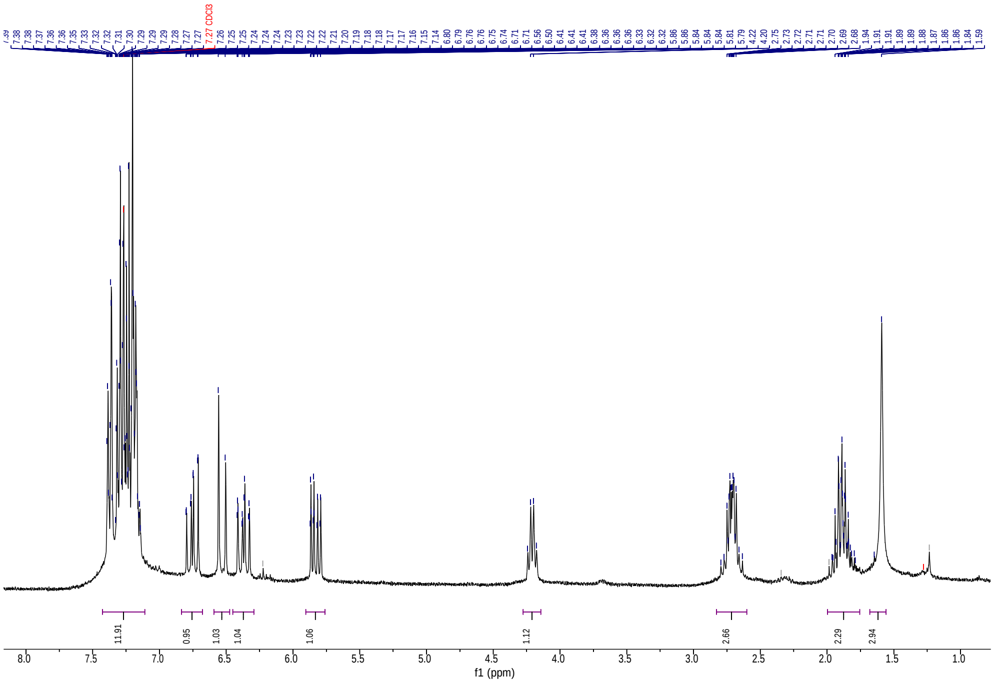

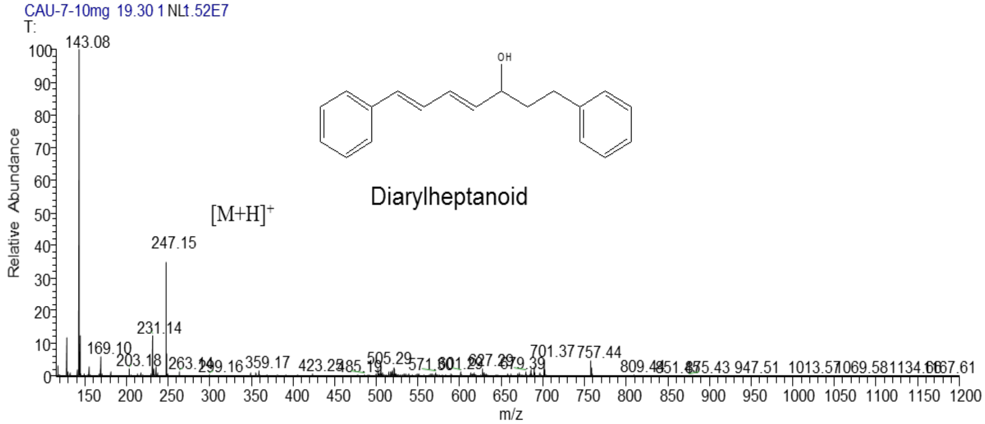
CAU-7-10mg 19.30 1 NL1.52E7
T:
143.08
247.15
231.14
169.10
203.18
[M+H]+
OH
Diarylheptanoid
Relative Abundance
m/z

germacrone (**5**)

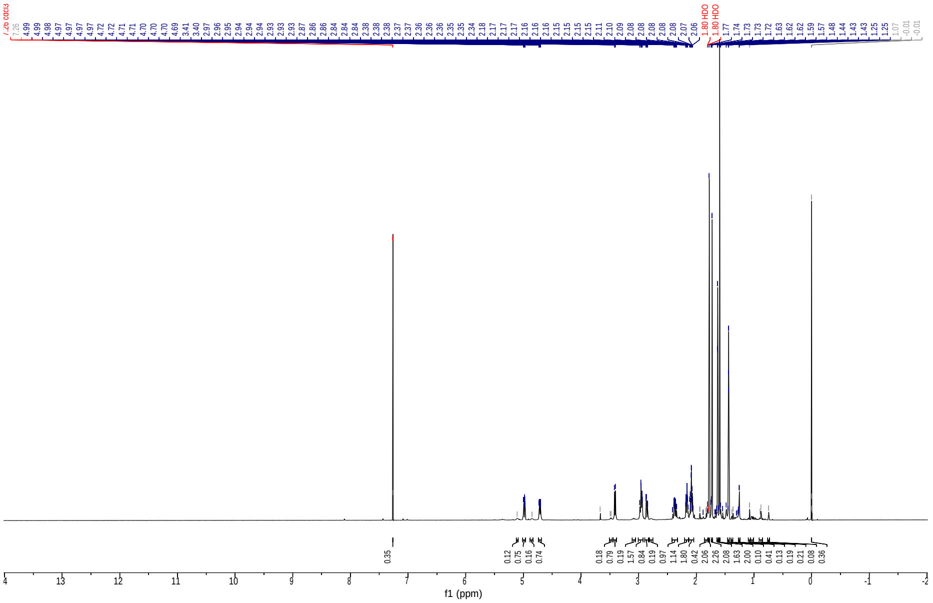

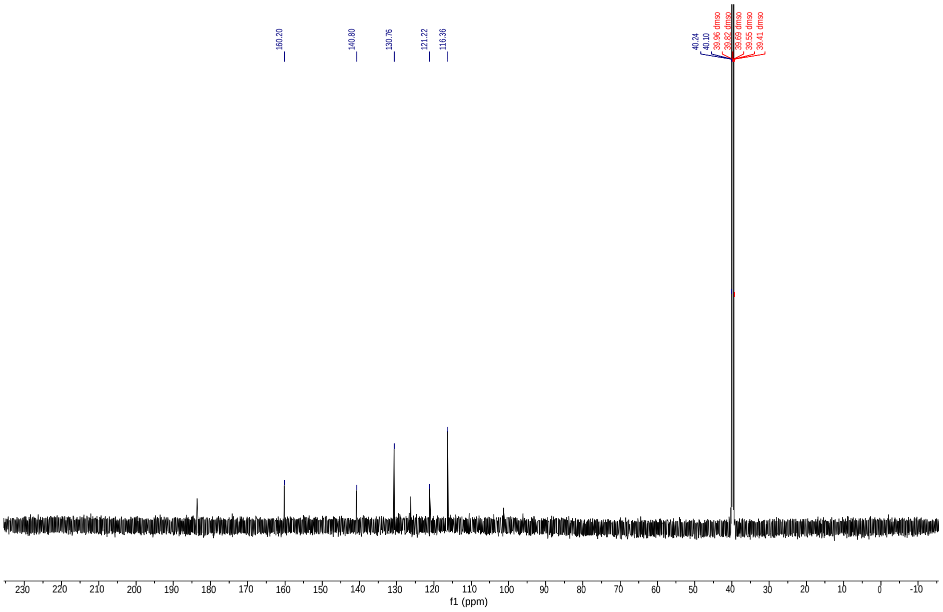

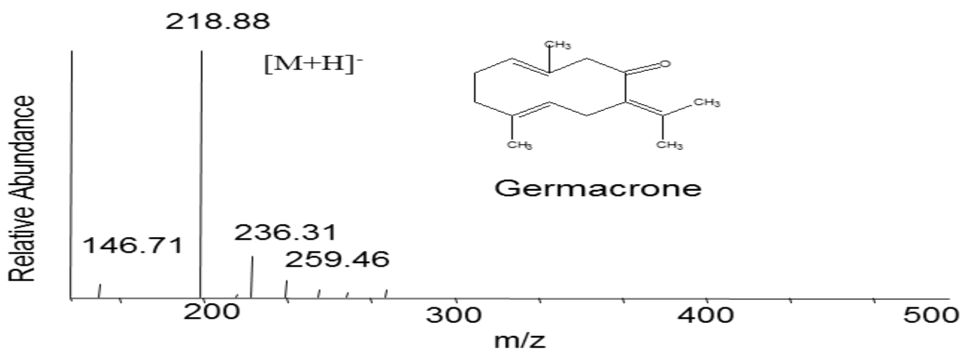

furanodienone (**6**)

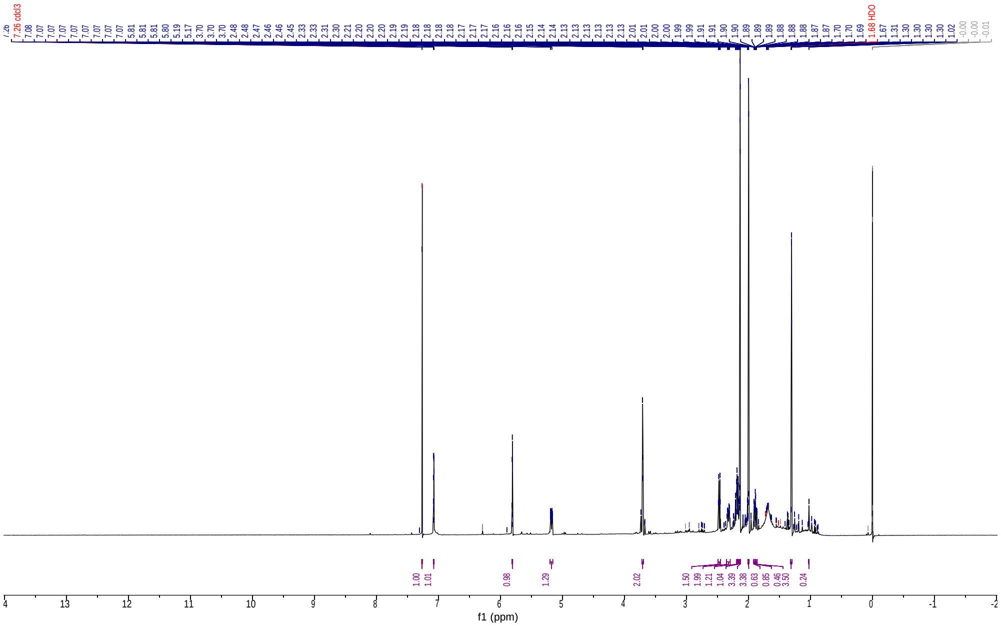

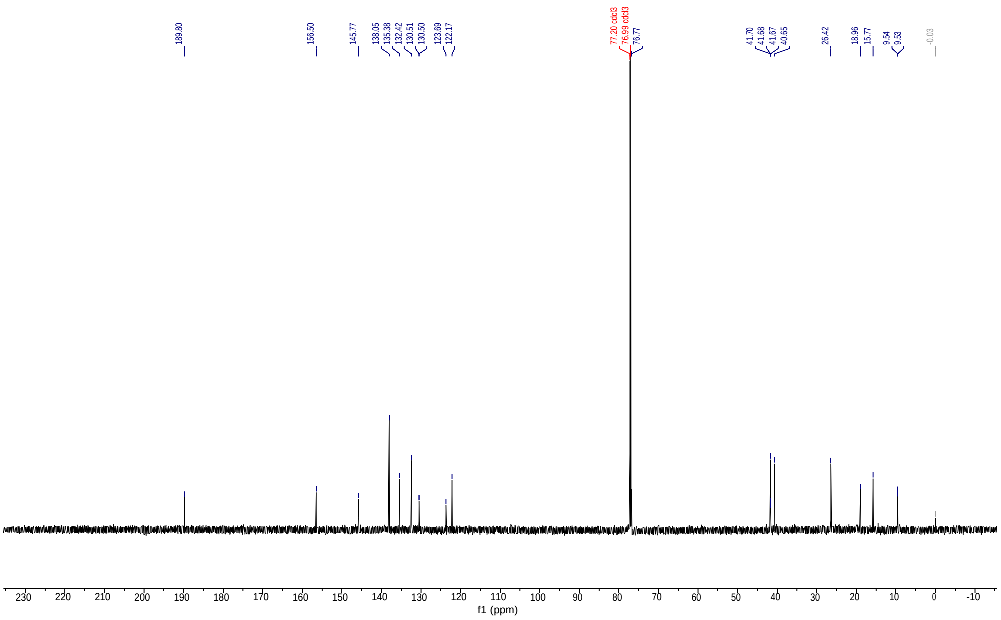

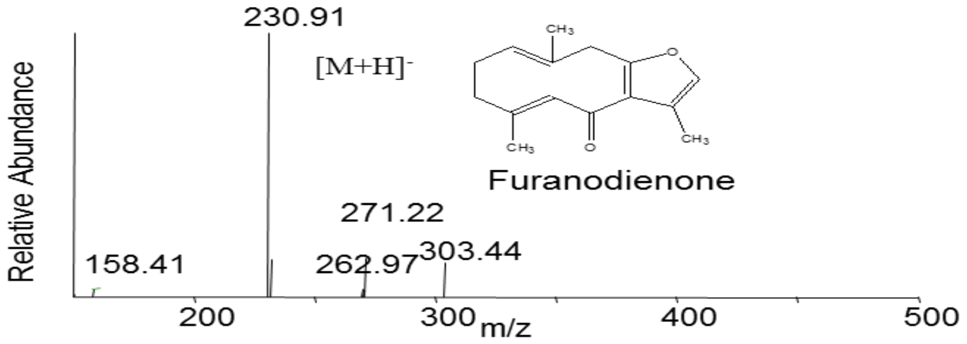

zederone (**7**)

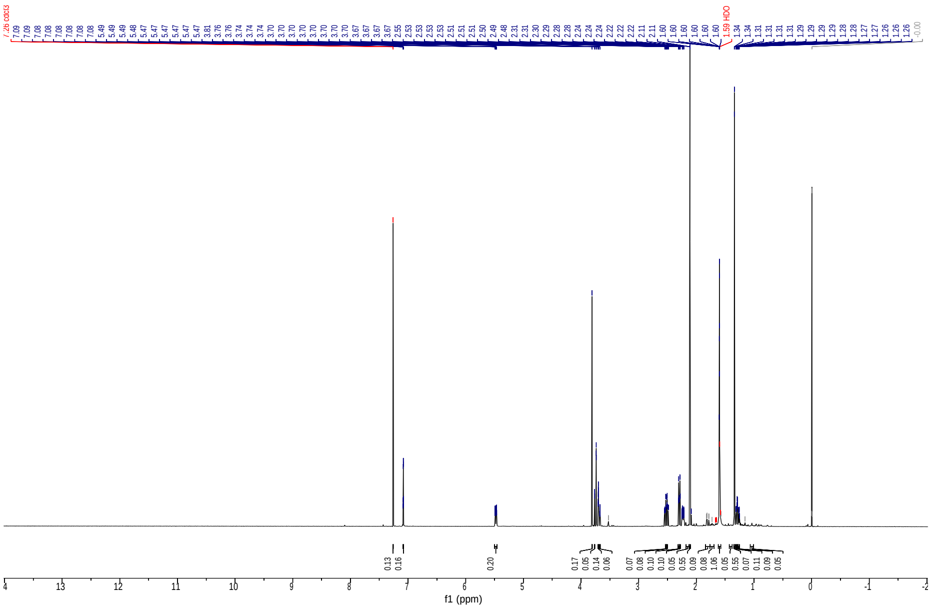

f1 (ppm)

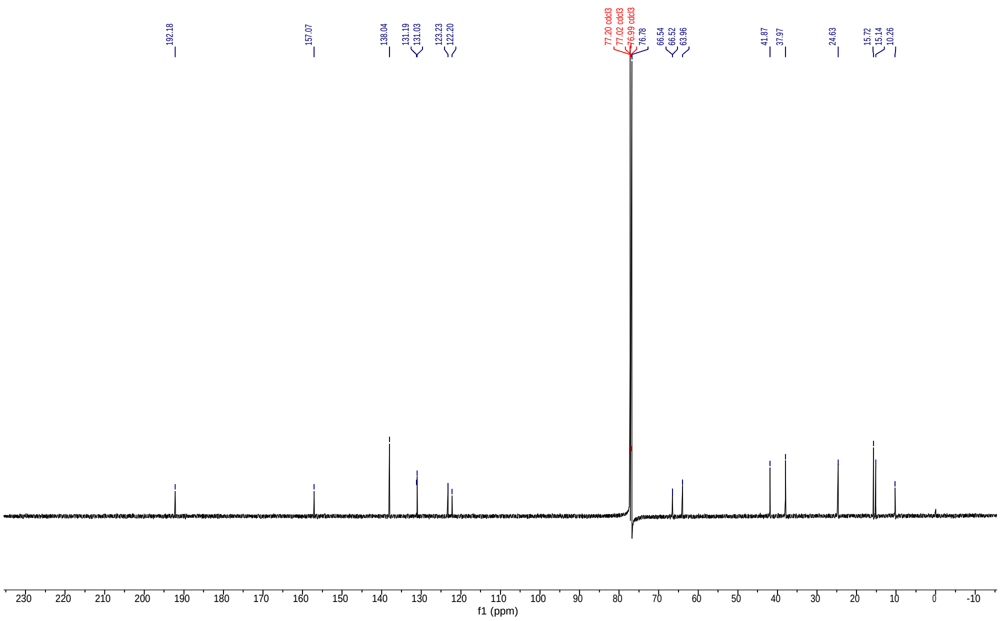

192.18
157.07
138.04
131.19
131.03
123.23
122.20
77.20 cdcl3
77.02 cdcl3
76.99 cdcl3
76.78
66.54
66.52
63.96
41.87
37.97
24.63
15.72
15.14
10.26
f1 (ppm)

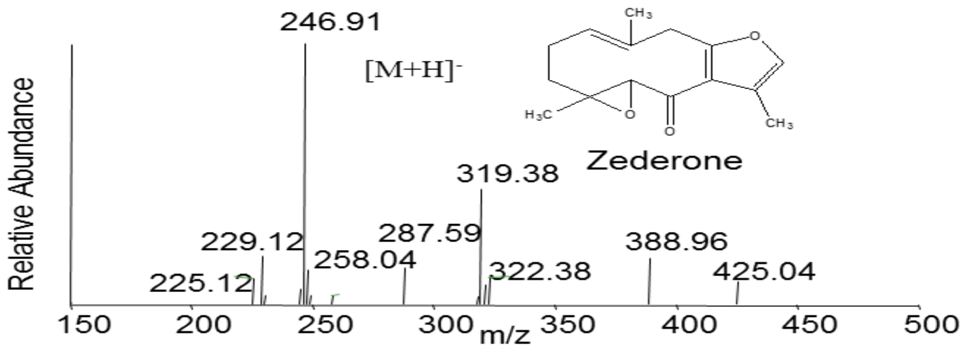

246.91
[M+H]-
319.38
229.12
287.59
258.04
225.12
322.38
388.96
425.04
Relative Abundance
m/z
Zederone

ar-turmerone (**8**)

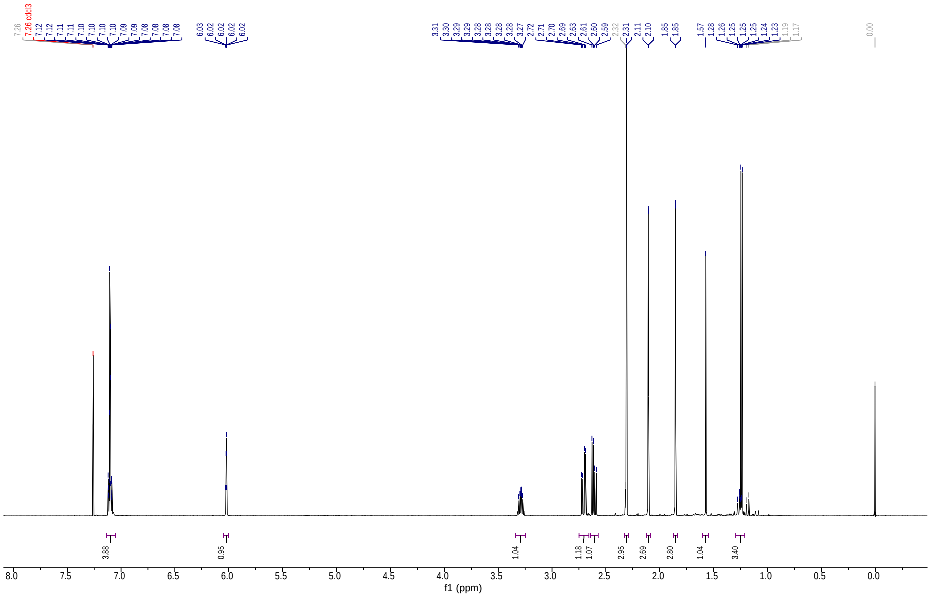

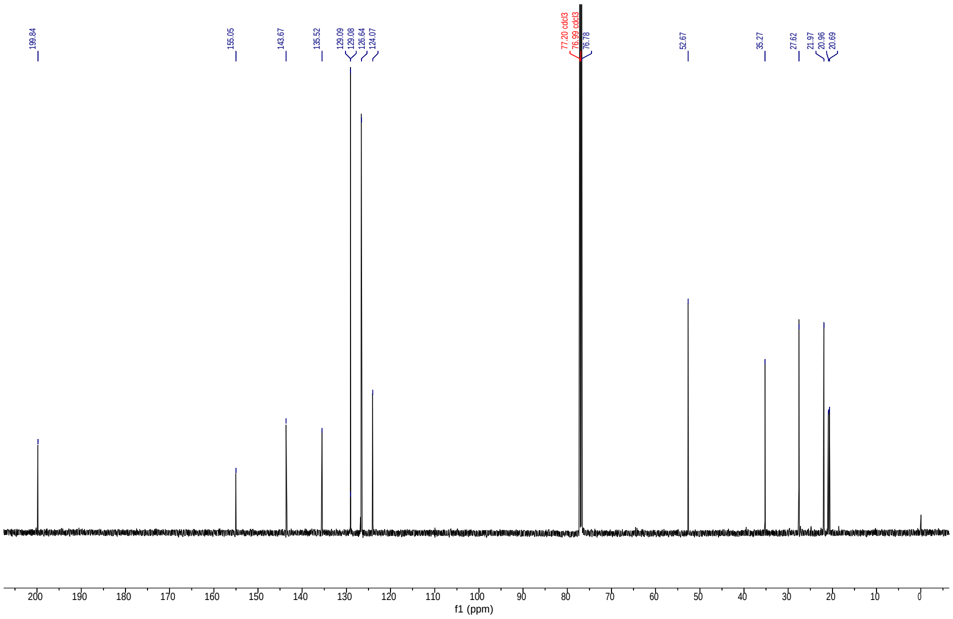

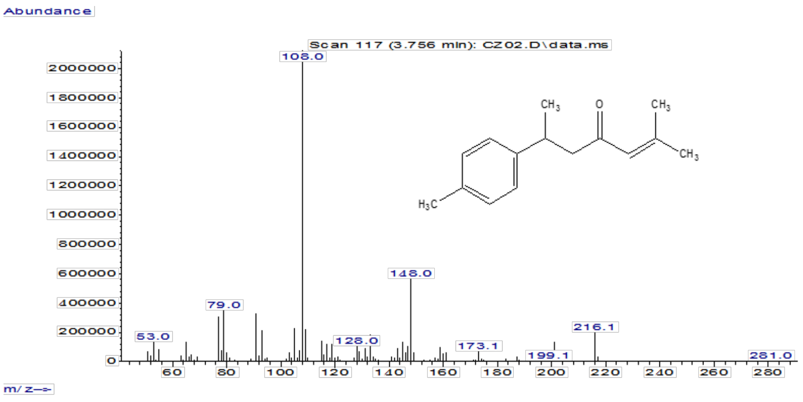

Taxonomy of *Curcuma* 23 species

- Sample: 23 *Curcuma* species
- Locality: Kanchanaburi province and Chatuchak market, Bangkok Thailand (2013), Plant collection at Tapong, Meaung, Rayong Thailand
- Herbarium deposit date and place: May and August 2015 at Bangkok Herbarium, Plant Variety Protection Division, Department of Agriculture, Bangkok Thailand

1. *Curcuma* 'Wan Ma-Leung'

Corm ovoid, pale yellow inside, 6-8 x 3-4 cm; **rhizome** branched, pale yellow inside. **Leafy shoot** 80-120 cm tall; **bladeless sheath** 5-30 cm long. **Leaf-sheath** glabrous; **ligule** membranous, glabrous, 2.5 mm long; **petiole** glabrous; **blade** lanceolate, glabrous, base cuneate, apex acuminate 50-80 x 12-16 cm. (no flower throughout this study)

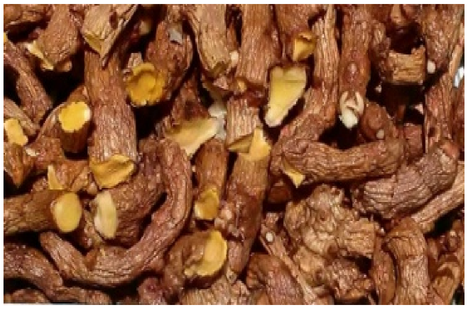

Thailand: Cultivated
Vernacular name: Wan Ma-Leung
Use: Folk medicine
Critical remarks:
Cultivated: MRG001, Bangkok Herbarium (BK), August 23, 2015

2. *Curcuma mangga* Val ex. Zijp 'Wan Khamin- Khao'

Corm white with yellow center, mango-scented, 3-4 x 2.3 cm; **rhizome** white with yellow center, 1.5 cm diameter; **leafy shoot** 60-100 cm tall. **Bladeless sheath; leaf-sheath** glabrous; **ligule** indistinct; **petiole** glabrous; **blade** green, glabrous on both surfaces, base cuneate, apex acute, 30-45 x 9-12 cm. **Inflorescence** lateral; **scape** 20 cm long, glabrous; **spike** 25-30 cm long. **Bracts** greenish white with green patch in center; **coma bracts** white with pink patch in center; **bracteole** obovate, folded, minute-hairy, apex acute, 25x15 mm. **Calyx** tubular, 8 mm long, minute-hairy, apex 3 lobed, split one side 3 mm. **Corolla tube** 25 mm long, minute-hairy; **lobes** 9-10 x 7-10 mm; dorsal one concave, hooded, minute-hairy, apex acute-mucronate; lateral ones shallowly concave, glabrous, apex rounded. **Staminodes** oblique obovate, glandular hairy, white, apex rounded, 12x8 mm. **Labellum** 3 lobed, mid-lobe bifid, glandular hairy long sides of mid-band, 12x14 mm. **Stamen** glabrous; filament flat, 2.5x3 mm; theca 4 mm long, crest less than 1 mm long, apex rounded; spurs flat, triangular, glabrous, point downwards, apex acuminate, 3

mm long. **Ovary** barrel-shaped, hairy, 3 mm long; **stylodes** cylindrical, glabrous, apex acute, 4 mm long; **stigma** bi-lobed, laterally opened, ciliate, 1 mm wide.

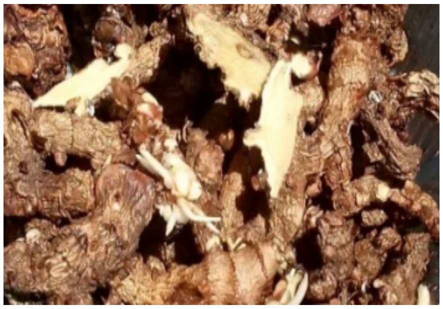

Thailand: Cultivated
Vernacular name: Wan Khamin- Khao
Use: The rhizomes are used as a stomachic and for chest pains, fever, and general debility. It is also used in postpartum care.
Critical remarks: This species has mango scented as in *Curcuma amada* Roxb. but different in produced lateral inflorescence.
Cultivated: MRG002, Bangkok Herbarium (BK), May 15, 2015

3. *Curcuma* 'Wan Ma-Hor'

Corm ovoid, pale brown, 6-8 x 4-5 cm; **rhizome** white inside. **Leafy shoot** 80-120 cm tall; **bladeless sheath** glabrous, 5-30 cm long. **Leaf sheath** glabrous, 30 cm long; **ligule** ciliate, 2.5 mm long; **petiole** short or sessile; **blade** lanceolate, base cuneate, apex acuminate. (no flower throughout this study)

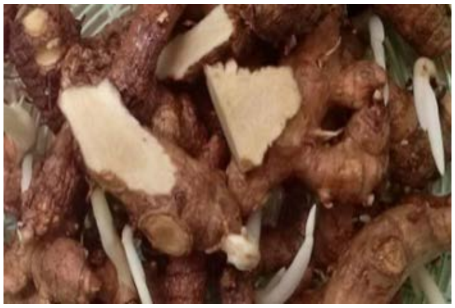

Thailand: Cultivated
Vernacular name: Wan Ma-Hor
Use: Folk medicine
Critical remarks:
Cultivated: MRG003, Bangkok Herbarium (BK), August 23, 2015

4. *Curcuma* 'Wan Khamin-Dam'

Corm ovoid, inside bluish black, 3.5 x 5 cm; **rhizome** branched, bluish black, **Leafy shoot** 80-100 cm tall; **bladeless sheath** green or reddish, glabrous, 5-30 cm long. **Leaf-sheath** glabrous,

green with red streak; **ligule** membranous; **petiole** glabrous; **blade** obovate-lanceolate, green with red patches on either sides of midrib, glabrous on both surfaces, base cuneate, apex acute, 30-45 x 5-15 cm. **Inflorescence** lateral; **spike** 15-20 cm long. **Bracts** green; **coma bracts** deep red which become crimson after maturation. **Calyx**10-15 mm long, obtuse and 3 toothed. **Corolla tube** long tubular, pale yellow lip; **Labellum** 3 lobed semi-elliptic.

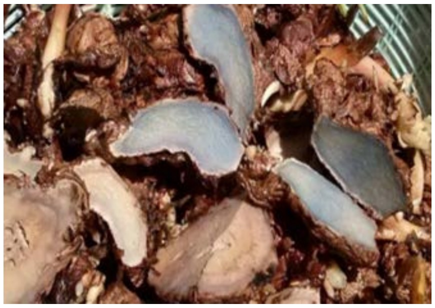

Thailand: Cultivated
Vernacular name: Wan Khamin-Dam
Use: Folk medicine
Critical remarks: The surface of rhizome is dark brown and bluish black
Cultivated: MRG004, Bangkok Herbarium (BK), August 23, 2015

5. *Curcuma* 'Wan Rang-Jud'

Corm ovoid, inside pale yellow, 4-8 x 3-5 cm; **rhizome** branched, pale yellow.**Leafy shoot** 80-100 cm tall; **bladeless sheath** green, glabrous, 10-40 cm long. **Leaf-sheath** glabrous, green; **ligule** membranous; **petiole** glabrous, 2-15 cm long; **blade** obovate-lanceolate, green, base cuneate, apex acute, 30-45 x 9-15 cm. **Inflorescence** lateral, 30-35 cm tall; scape 15-20 cm long. **Bracts** obovate, apex acute, 4 x2.5 cm; **coma bracts** obovate; **bracteoles** boat-shaped, apex rounded. **Corolla tube** 20 mm long, lower half white, upper half pink; **lobes** pink, 10 x13 mm; **dorsal lobe** concave, hooded, apex acute-mucronate. **Staminodes** broad obovate, glandular hairy on inner surface, apex truncate. **Labellum** 3 lobed, 20x20 mm, glandular hairy on inner surface, denser along either side of midband, side-lobes rounded, mid-lobe emarginate. **Filament** scattered hairy, anther, hairy, pollen sac 3.5 mm long; spurs sharply acute, downward-pointed, 2.5 mm long. **Ovary** barrel-shaped, 4.5 mm long, hairy on upper half; apex acute; stigma 1 mm wide, laterally opened, ciliate.

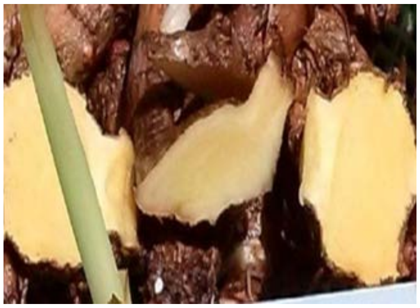

Thailand: Cultivated

Vernacular name: Wan Rang-Jud
Use: Folk medicine and antidote
Critical remarks:
Cultivated: MRG005, Bangkok Herbarium (BK), August 23, 2015

6. ***Curcuma aeruginosa*** **Roxb. 'Wan Maha-Mek'**

Corm ovoid, inside bluish green, 4-7 x 3-5 cm; **rhizome** branched, bluish green, 2 cm thick, **Leafy shoot** 80-90 cm tall; **bladeless sheath** green or reddish, glabrous, 8-30 cm long. **Leaf-sheath** glabrous, green with red streak; **ligule** membranous, bi-lobed, 2 mm long; **petiole** glabrous, 1-15 cm long; **blade** obovate-lanceolate, green with red patches on either sides of midrib, glabrous on both surfaces, base cuneate, apex acute, 35-45 x 9-12 cm. **Inflorescence** lateral, 30-35 cm tall; scape 15-20 cm long. **Bracts** obovate, short hairs on both surfaces, apex acute, 4.5x2.5 cm; **coma bracts** narrowly obovate to lanceolate, short hair on both surfaces, 5x1.5 cm; **bracteoles** boat-shaped, minute-hairy, apex rounded, with few hairs on ridge at tip, 16x10 mm. **Corolla tube** 25 mm long, minute-hairy, hair-ring in throat, lower half white, upper half red; **lobes** red, 13x10-12 mm; **dorsal lobe** concave, hooded, apex acute-mucronate, hairy at tip; **lateral lobes** shallowly concave, apex rounded, glabrous. **Staminodes** rectangular, pale yellow, glandular hairy, apex truncate, 12x8 mm. **Labellum** 3-lobed, mid-lobe retuse, glandular hairy along side of yellow mid-band, 16-18 mm. **Filament** flat, 3x4 mm; **theca** 4.5 mm long; **crest** less than 1 mm long, apex rounded; **spurs** flat, triangular, point downwards, apex acuminate, 2.5 mm long. **Ovary** barrel-shaped, densely hairy, 4 mm long; **stylodes** cylindrical, apex acute, 5-6 mm long; **stigma** 2-lobed, laterally opened, ciliate, 1.5 mm wide.

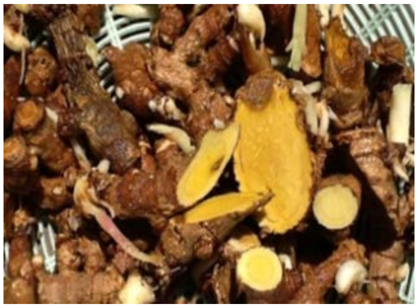

Thailand: Cultivated
Vernacular name: Wan Maha-Mek
Use: The rhizome of this plant is used medicinally to treat asthma and cough, scurvy and mental derangements
Critical remarks: This species is easily recognized by its bluish green rhizome, as suggested by epithet. The other species with color of rhizome look alike is *Curcuma caesia* which is bluish gray.
Cultivated: MRG006, Bangkok Herbarium (BK), August 27, 2015

7. ***Curcuma comosa*** **Roxb. 'Wan Chak- Mod- Luk'**

Corm ovoid, pale brown to white inside, 5-6 x 8-10 cm; **rhizome** very short. **Leafy shoot** 60 tall. **Leaf-sheath** 25-40 cm long; **ligule** indistinct; **petiole** 0-5 cm long; **blade** green with red patches on wither sides of mid-vein when young, glabrous on both surfaces, base cuneate, apex acute, 2-40 x 14-17 cm. **Inflorescence** lateral; **scape** 10 cm long, glabrous; **spike** 20 cm long. **Bracts** ovate, white with green patch in center, tipped with pink, glabrous on both surfaces; coma bracts pinkish, apex acute. **Bracteoles** broadly obovate, glabrous, apex acute to retuse, 18x15 mm. **Calyx** tubular, glabrous, 8 mm long, apex 3-lobed, split one side 4 mm. **Corolla** tube white, glabrous, 25 mm long; lobes white, glabrous, 9 x 5-8 mm; dorsal one concave, hooded, apex acute-mucronate, with few hair on tip; lateral ones shallowly concave, apex rounded. **Staminodes** broadly obovate, margin irregular lobed, glandular hairy, 10x7 mm. **Labellum** obovate, 3-lobed, mid-lobed retuse, glandular hairy along sides of yellow mid-brand, 10x12 mm. **Filament** flat, glabrous, 3x3 mm; anther glabrous; theca 4 mm long; spurs flat, triangular, acuminate, 2 mm long; crest less than 1 mm long, apex rounded. **Ovary** barrel-shaped, glabrous, 4 mm long; **stylodes** cylindrical, acute, 5 mm long; **stigma** 2-lobed, laterally opened, ciliate.

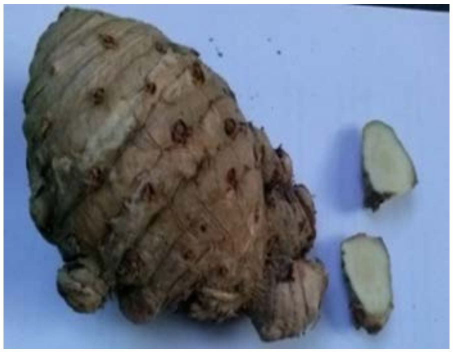

Thailand: Cultivated

Vernacular name: Wan Chak- Mod- Luk (ตัวเมีย)

Use: Rhizomes, used medically treat postpartum uterine bleeding and as an aromatic stomachic. Biological properties, such as oestrogenic (Winuthayanon, Piyachaturawat et al., 2009; Winuthayanon, Suksen et al., 2009), nematocidal (Jurgens et al., 1994), anti-inflammatory (Jantaratnotai, Utaisincharoen, Piyachaturawat, Chongthammakun, & Sanvarinda, 2006), and choloretic (Piyachaturawat, Charoenpiboonsin, Toskulkao, & Suksamrarn, 1999) activities.
Critical remarks: This species produces subsessile inflorescence. Bracts white tipped with pink
Cultivated: MRG007, Bangkok Herbarium (BK), August 23, 2015

8. ***Curcuma*** **'Wan Kanta- Mala'**

Corm ovoid, pale yellow inside, 4-10 x 3-7 cm; **rhizome** branched, pale yellow. **Leafy shoot** 80-90 cm tall; **bladeless sheath** green or reddish, glabrous, 8-30 cm long. **Leaf-sheath** glabrous, green with red streak; **ligule** membranous, bi-lobed, 2 mm long; **petiole** glabrous, 1-15 cm long; **blade** obovate-lanceolate, green with red patches on either sides of midrib, glabrous on both surfaces, base cuneate, apex acute, 30-45 x 5-10 cm. (no flower throughout this study)

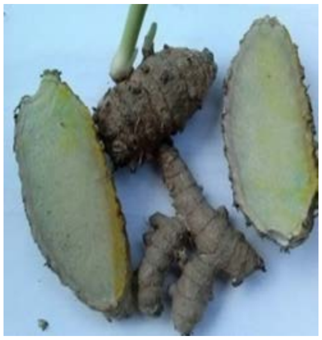

Thailand: Cultivated
Vernacular name: Wan Kanta- Mala
Use: Folk medicine
Critical remarks:
Cultivated: MRG008, Bangkok Herbarium (BK), August 27, 2015

9. ***Curcuma aurantiaca*** **Van Zijp. 'Wan En-Leung'**

Corm ovoid, pale yellow inside, 4x3 cm; **rhizome** very short; **leafy shoot** 30-50 cm tall. **Bladeless-sheath** 3-4, green, hairy, apex mucronate, 5-25 cm long. **Leaf-sheath** green, hairy, 12-25 cm long; **ligule** 2-lobed, densely hairy, ciliate, lobe apices obtuse, 3-6 mm long; **petiole** hairy, 5-20 cm long; **blade** obovate to lanceolate, green, lower surface pubescent, upper one hairy along secondary nerves, base broad cuneate to slightly cordate, apex acuminate or caudate, 15-45 x 10-15 cm. **Inflorescence** terminal; **scape** densely hairy, 10-15 cm long; **spike** 9-20 cm long. **Bracts** ovate, green, pubescent on both surface, apex rounded to obtuse, 3-7 x 2-4 cm; **coma bracts** lanceolate, pink, pubescent on both surface, apex rounded, 3-7 x 1.2-2 cm. **Bracteoles** triangular, hairy, apex obtuse, 7x7 mm. **Calyx** tubular 12 mm long, hairy, split one side 5 mm, apex 3-lobed, lobe apices rounded to obtuse. **Corolla tube** glabrous, 25 mm long; **lobes** 7-10 x 15 mm; dorsal one hooded, hirtellous (softly or minutely hirsute), apex mucronate; lateral ones shallowly concave, glabrous, apex obtuse. **Staminodes** obliquely obovate, sparsely minute-hairy, apex rounded to truncate, 12-25 x 9-10 mm. **Labellum** 3 lobed, mid-lobe apex bifid, hairy along both sides of mid-band, 18x18 mm. **Filament** flat, glabrous, 3x3 mm; theca 6 mm long; spur absent, crest 1 mm long, grooved. **Ovary** barrel-shaped, sericeous, 4 mm long; **stylodes** 4 mm long; **stigma** 2-lobed, laterally opened, 2 mm wide.

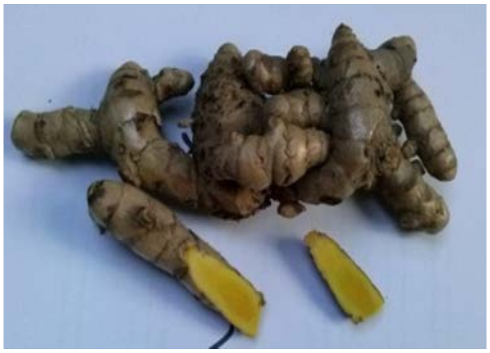

Thailand: Cultivated
Vernacular name: Wan En-Leung
Use: Folk medicine
Critical remarks: Flower orange yellow.
Cultivated: MRG009, Bangkok Herbarium (BK), August 23, 2015

10. ***Curcuma aromatica*** **Salisb. 'Wan Nang-Kam'**

Corm avoid, bright yellow inside, 5x3 cm; **rhizome** branched, bright yellow inside. **Leafy shoot** 80-100 cm tall; **bladeless sheath** short hairy, 12-27 cm long. **Leaf-sheath** green, short hairy, 45-55 cm long; **ligule** membranous, 1.5 mm long, **petiole** glabrous, 0-5 cm long; **blade** oblanceolate, green with red patch along midrib, glabrous on both surfaces, base cuneate, apex acuminate. **Inflorescence** lateral, 30 cm tall; scape short hairy, 15 cm long. **Bracts** elliptic, short hairy on both surfaces, apex acute, 4.0-4.5 x 2.5-3.0 cm; **bracteoles** broad elliptic, 15-17 x 13 mm, hairy along ridges, apex obtuse. **Calyx** tubular 8 mm long, hairy at base and along ridges, split down one side 4 mm, apex shallowly 3-lobed. **Corolla** tube 27 mm long, glabrous; corolla lobes 13-15 x 7-8 mm, glabrous; dorsal one hooded, acute-mucronate, hairy at tip, lateral ones; slightly concave, apex obtuse. **Staminodes** broad obovate, glandular hairy on inner surface, apex truncate. **Labellum** 3 lobed, 20x20 mm, glandular hairy on inner surface, denser along either side of midband, side-lobes rounded, mid-lobe emarginate. **Filament** scattered hairy, anther, hairy, pollen sac 4 mm long; spurs sharply acute, downward-pointed, 2.5 mm long. **Ovary** barrel-shaped, 4 mm long, hairy on upper half; apex acute; stigma 1 mm wide, laterally opened, ciliate.

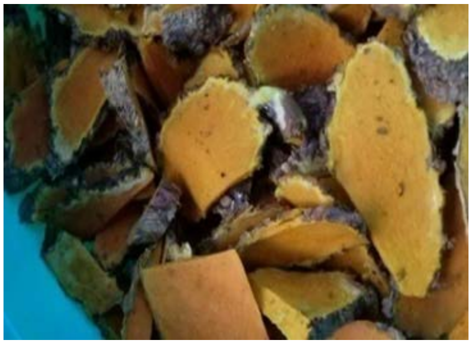

Thailand: Cultivated
Vernacular name: Wan Nang-Kam
Use: As an aromatic for cosmetic purposes and in indigenous medicine for external applications on skin diseases, bruises and sprains
Critical remarks: Rhizome of this species is bright yellow
Cultivated: MRG010, Bangkok Herbarium (BK), May15, 2015

11. ***Curcuma latifolia*** **Rosc. 'Wan Chak- Mod- Luk'**

Corm ovoid, pale brown inside, 10-12 x 5-8 cm; **rhizome** short. **Leafy shoot** 150-200 cm tall; **bladeless sheath** 20-30 cm long. **Leaf sheath** 50-80 cm long; **ligule** membranous, truncate, 5 mm long; **petiole** 5-20 cm long, sparsely short hairy; **blade** almost glabrous on both surfaces, except along midrib and margin on lower surface, green with reddish brown patch along midrib, base cuneate, apex acuminate. **Inflorescence** terminal or lateral; **scape** 20 cm long; **spike** 15 cm long; **lateral inflorescence** 25-35 cm tall; **scape** 12-20 cm long, hairy, **sheath** up to 15 cm long, hairy. **Bracts** 3.5-6 x 3 cm, with short hairy on both surfaces, apex rounded to obtuse; **coma** bracts 6x3 cm, with short hairs on both surfaces, apex rounded or obtuse; coma bracts 4x2.5 cm, hairy on both surfaces, apex acute to obtuse; **bracteoles** concave, glabrous, apex rounded or obtuse. **Calyx** tubular, 10 mm long, split one side 6 mm, few hairs along veins, apex shallowly 3-lobed. **Corolla tube** glabrous, 20 mm long; **lobes** glabrous; **dorsal** one hooded, apex acute mucronate; **lateral** ones concave, apex rounded. **Staminodes** obovate, glandular hairy. **Labellum** 3-lobed, midlobe emarginate, yellow with bright yellow midband, glandular hairy along side of midband, 13x15 mm. **Filament** flat, 3x3 mm; **anther** 4 mm long; **spurs** sharply acute. **Ovary** barrel-shaped, densely hairy, 4 mm long; **stylodes** cylindrical, 4 mm long; **stigma** laterally opened, ciliate.

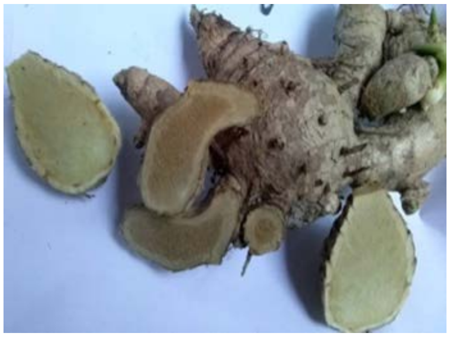

Thailand: Cultivated

Vernacular name: Wan Chak- Mod- Luk (ตัวผู้)

Use: Used as an aromatic stomachic and an indigenous anti-inflammatory medicine.
Critical remarks: This is the largest species of *Curcuma* found in Thailand
Cultivated: MRG011, Bangkok Herbarium (BK), August 23, 2015

12. *Curcuma zedoaria* Rosc. 'Wan Khamin-Oil'

Corm ovoid, pale brown, 6-8 x 4-5 cm; **rhizome** short. **Leafy shoot** 80-120 cm tall; **bladeless sheath** glabrous, 5-20 cm long. **Leaf sheath** glabrous, 28 cm long; **ligule** ciliate, 2 mm long; **petiole** very short or sessile; **blade** lanceolate, base cuneate, apex acuminate. **Inflorescences** lateral; **scape** glabrous, 18-22 cm long; **spike** 10-14 cm long. **Bracts** broadly ovate, glabrous, green with pink tip, apex obtuse to acute, 4-4.5x 3 cm; **coma bracts** ovate, reddish pink, apex acute to broadly acute, 6x2 cm; **bracteoles** ovate, shallowly concave, hairy on outer surface, apex obtuse, 7x3.5 mm. **Calyx** tubular, 6 mm long, split down one side 3 mm, hairy, apex shallowly 3-lobed. **Corolla tube** hairy on upper half, 20 mm long; **lobes** glabrous, 8-9 x 6-9 mm;

dorsal one hooded; lateral ones shallowly concave, apex rounded. **Staminodes** obliquely obovate, yellow, glandular hairy, apex rounded to truncate, 11x5 mm. **Labellum** 3-lobed, yellow with bright yellow mid-band, hairy along sides of mid-band, midlobe emarginate, 9x11 mm. **Filament** flat, anther 2.5 mm long; spurs sharply acute. **Ovary** barrel-shaped, pubescent, 3 mm long; **stylodes** cylindrical, 3 mm long; **stigma** 2-lobed, laterally opened, ciliate, 1 mm wide.

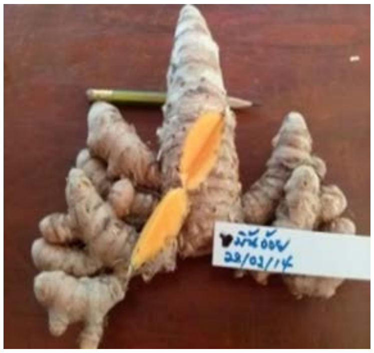

Thailand: Cultivated

Vernacular name: Wan Khamin-Oil

Use: It is used traditionally for the treatment of menstrual disorders, dyspepsia, vomiting and cancer.

Critical remarks: Bright red coma

Cultivated: MRG012, Bangkok Herbarium (BK), May 15, 2015

13. *Curcuma longa* L. 'Wan Khamin-Chan'

Corm ovoid, orange yellow inside, 6-8 x 3-4 cm; **rhizome** branched, orange yellow inside, 1.5-2 cm. **Leafy shoot** 80-120 cm tall; **bladeless sheath** 10-25 cm long. **Leaf-sheath** glabrous, 10-30 cm long; **ligule** membranous, glabrous, 3 mm long; **petiole** glabrous, 5-8 cm long; **blade** lanceolate to elliptic, glabrous on both surfaces, base cuneate, apex acuminate, 65-80 x 12-16 cm. **Inflorescences** terminal, 25-30 cm long; **scape** 10-15 cm long; **spike** long. **Bracts** oblong to obovate, pubescent on both surfaces, green, apex broadly acute to rounded; **coma bracts** lanceolate, pubescent on both surfaces, pure white or white tipped with pale pink; **bracteoles** obovate, keeled. **Calyx** tubular, 10 mm long, split down one side 4 mm, glabrous, apex shallowly 3-lobed. **Corolla tube** glabrous, 32 mm long; lobes 13-14 x 8-10 mm; dorsal lobe hooded; lateral lobes concave, apex rounded. **Staminodes** oblong, pale yellow, apex rounded or truncate, 13x9 mm. **Labellum** 3-lobed, mid lobe emarginate, pale yellow with yellow midband, 22x16 mm. **Filament** 5x3 mm; **anther** 3.5 mm long; **spurs** sharply acute. **Ovary** barrel-shaped, hairy, 4 mm long; **stylodes** 3.5 mm long; **stigma** 2-lobed, laterally opened, ciliate, 1 mm wide.

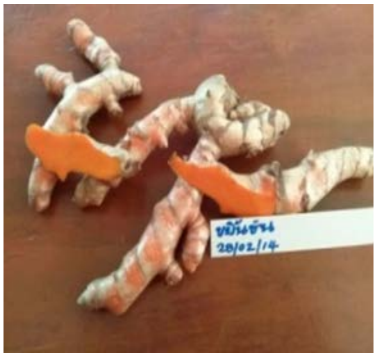

Thailand: Cultivated
Vernacular name: Wan Khamin-Chan
Use: Spice and medicinal plant
Critical remarks:
Cultivated: MRG013, Bangkok Herbarium (BK), August 23, 2015

14. *Curcuma* 'Wan Muang'

Corm white with pale yellow, 3-5 x 2.5 cm; **rhizome** white with pale yellow, 2 cm diameter; **leafy shoot** 50-80 cm. **Bladeless sheath; leaf-sheath** glabrous; **ligule** indistinct; **petiole** glabrous; **blade** green, glabrous on both surfaces, base cuneate, apex acute, 30-45 x 9-12 cm. (no flower throughout this study)

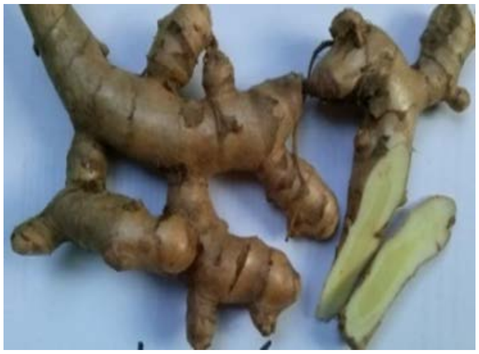

Thailand: North and Northeast
Vernacular name: Wan Muang
Use: Folk medicine
Critical remarks:
Cultivated: MRG014, Bangkok Herbarium (BK), August 23, 2015

15. *Curcuma angustifolia* Roxb. 'Wan Khai-Khun'

Corm ovoid, pale brown to white inside, 3-5 x 3-4 cm; **rhizome** very short. **Leafy shoot** 20-70 cm tall; **bladeless-sheath** 3-15 cm long, dense short hairy, ciliate. **Leaf** sheath 10-40 cm long, hairy, ciliate; **ligule** 2-lobed, 2 mm long, hairy, ciliate; **petiole** 10 cm long, glabrous or hairy; **blade** 25-60 x 3-6.5 cm, glabrous or pubescent on both surface, base attenuate, apex acuminate.

Inflorescence terminal or lateral; scape 5-20 cm long, hairy; **spike** 10-12 cm long; **bracts** glabrous or hairy, apex rounded or obtuse; **coma bracts** 30-40 x 7-10 cm, hairy, apex acute or obtuse; **bracteole** triangular, 8x8 mm, sparely hairy or glabrous, apex obtuse to acute. **Calyx** tubular, 7-8 long, glabrous, split down one side 3-4 mm long, apex 3 lobed, with ridges from base of each lobe and ended by small beak-like structure just below the apex of lobe; **corolla tube** 20-25 mm long, glabrous outside, hairy inside; **corolla lobes** 6x10 mm, glabrous; dorsal one hooded, apex acuminate, with few hairs; lateral one shallowly concave, apex rounded. **Staminodes** obovate, with short hairs at the middle of apical half, apex rounded or truncate; **labellum** 3-lobed, hairy at base and along either side of mid-band, mid-lobed emarginate. **Filament** flat, glabrous or sparsely short hairy; **anther** 3-4 x 2 mm, glabrous; **spurs** flat, triangular, 1-2 mm long, apex acuminate; **Ovary** barrel-shaped, 2-3 mm long, hairy; **stylodes** cylindrical or clavate, 3-4 mm long, apex obtuse; **stigma** 1 mm wide, ciliate, open laterally.

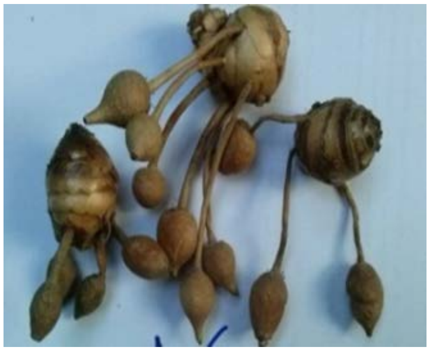

Thailand: North, Northeast and Southwest
Vernacular name: Wan Khai-Khun
Use: Young inflorescence for vegetable
Critical remarks: It is most variable species and has widest range of distribution
Cultivated: MRG015, Bangkok Herbarium (BK), August 23, 2015

16. *Curcuma* 'Wan Khabitong'

Corm ovoid, yellow inside, 5-10 x 3-5 cm; **rhizome** branched, yellow inside. **Leafy shoot** 80-100 cm tall; **bladeless sheath** 5-30 cm long. **Leaf-sheath** glabrous; **ligule** membranous, glabrous, 2.5 mm long; **petiole** glabrous; **blade** lanceolate, glabrous, base cuneate, apex acuminate 60-80 x 12-15 cm. (no flower throughout this study)

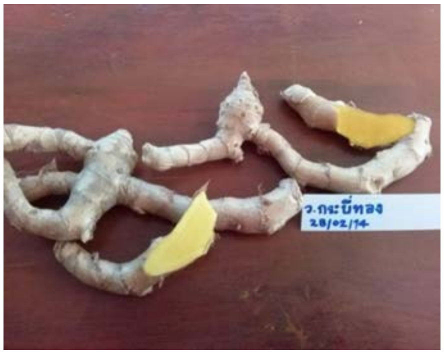

Thailand: Cultivated
Vernacular name: Wan Khabitong
Use: Folk medicine
Critical remarks:
Cultivated: MRG016, Bangkok Herbarium (BK), August 23, 2015

17. ***Curcuma*** **'Wan Pataba'**

Corm ovoid, 3-6 x 3 cm; **rhizome** white pale yellow inside, 2 cm diameter; **leafy shoot** 60-100 cm tall. **Bladeless sheath; leaf-sheath** glabrous; **ligule** indistinct; **petiole** glabrous; **blade** almost glabrous on both surfaces, except along midrib and margin on lower surface, green with reddish brown patch along midrib, apex acuminate, 30-55 x 9-15 cm. (no flower throughout this study)

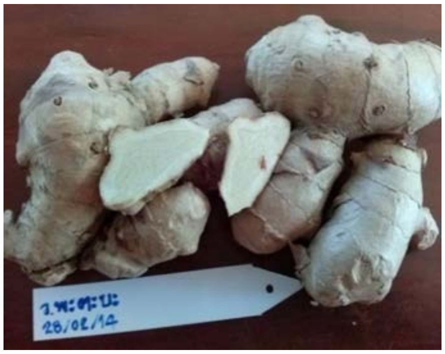

Thailand: Cultivated
Vernacular name: Wan Pataba
Use: Folk medicine
Critical remarks:
Cultivated: MRG017, Bangkok Herbarium (BK), August 23, 2015

18. ***Curcuma*** **'Wan Kortong'**

Corm ovoid, yellow inside, 5-12 x 3-5 cm; **rhizome** branched, yellow inside. **Leafy shoot** 80-120 cm tall; **bladeless sheath** 5-30 cm long. **Leaf-sheath** glabrous; **ligule** membranous, glabrous, 3 mm long; **petiole** glabrous; **blade** lanceolate, glabrous, base cuneate, apex acuminate 60-80 x 12-15 cm. (no flower throughout this study)

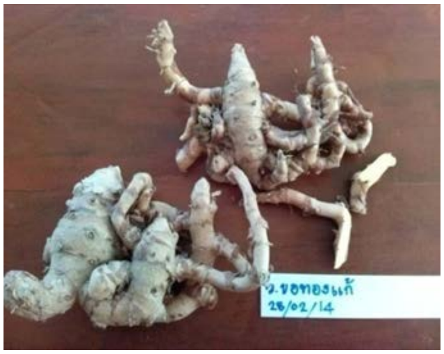

Thailand: Cultivated
Vernacular name: Wan Kortong
Use: Folk medicine
Critical remarks:
Cultivated: MRG018, Bangkok Herbarium (BK), August 23, 2015

19. ***Curcuma*** **'Wan Na-Natong'**

Corm ovoid, pale yellow inside, 3-10 x 2-5 cm; **rhizome** branched, pale yellow inside. **Leafy shoot** 80-100 cm tall; **bladeless sheath** 5-30 cm long. **Leaf-sheath** glabrous; **ligule** membranous, glabrous, 2-2.5 mm long; **petiole** glabrous; **blade** lanceolate, glabrous, base cuneate, apex acuminate 40-60 x 10-13 cm. (no flower throughout this study)

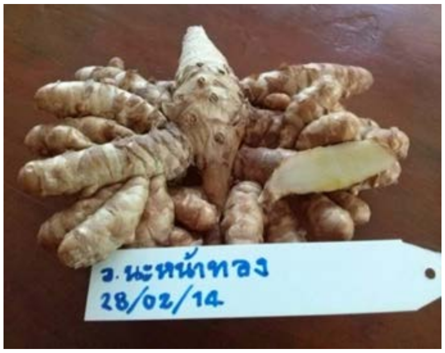

Thailand: Cultivated
Vernacular name: Wan Na-Natong
Use: Folk medicine
Critical remarks:
Cultivated: MRG019, Bangkok Herbarium (BK), August 23, 2015

20. ***Curcuma petiolata*** **Roxb. 'Wan Mahachakkapad'**

Corm ovoid, bright yellow inside, 3-5 x 5-8 cm; **rhizome** short; **leafy shoot** 35-80 cm tall. **Leaf sheath** 20-30 cm long; **ligule** 4 mm long, sparsely hairy; **petiole** 8-25 cm long, glabrous; **blade** 35-45 x 16-22 cm, glabrous on both surfaces or with few hairs along the secondary nerves, base rounded to broad cuneate, apex acuminate. **Inflorescence** terminal; **scape** 22-35 cm long, densely pubescent; **spike** 13-20 cm long. **Bracts** 4-5.5 x 1.5-2 cm, pilose on both surfaces, apex rounded to truncate or slightly emarginate; **coma bracts** 4-4.5 x 2-2.5 cm, pilose on both surfaces, apex obtuse to acute. **Calyx** 8 mm long, split down one side 3 mm, glabrous, apex 3-lobed; **corolla tube** 2 cm long, glabrous outside, with dense long hair ring inside just below the throat; **corolla lobes** 12-15 x 6-8 mm, glabrous, dorsal one hooded, apex acute, lateral ones slightly concave, apex obtuse. **Staminodes** obovate, 12x8 mm, sparsely glandular hairy in the middle, apex obtuse; **labellum** 3-lobed, 12x15 mm, sparsely glandular hairy along mid-band and in throat, apex of mid-lobe emarginate. **Filament** concave, 3x5 mm, glabrous; **anther** oblong,

glabrous; **spurs** triangular, 2 mm long; **crest** quadrangular, concave, apex rounded, covering half of stigma. **Ovary** barrel-shaped, 3 mm long, hairy; **stylodes** clavate, 3 mm long, apex rounded; **stigma** 1 mm wide, ciliate, open laterally.

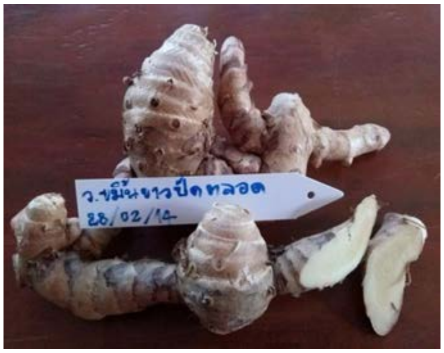

Thailand: Cultivated
Vernacular name: Wan Mahachakkapad
Use: Folk medicine
Critical remarks: The bract with rounded apex and metallic pink color are distinguished character.
Cultivated: MRG020, Bangkok Herbarium (BK), August 23, 2015

21. *Curcuma* 'Wan Khamin-Khao-Padtalod'

Corm pale yellow 3-6 x 3 cm; **rhizome** white yellow inside, 2 cm diameter; **leafy shoot** 60-120 cm tall. **Bladeless sheath; leaf-sheath** glabrous; **ligule** indistinct; **petiole** glabrous; **blade** almost glabrous on both surfaces, except along midrib and margin on lower surface, green with reddish brown patch along midrib, apex acuminate, 30-55 x 9-15 cm. (no flower throughout this study)

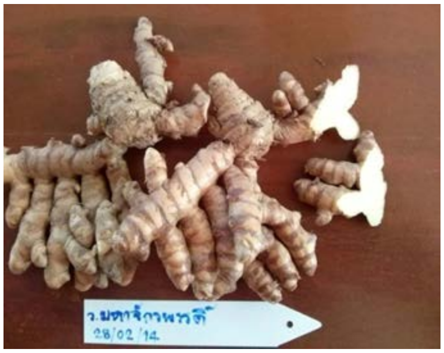

Thailand: Cultivated
Vernacular name: Wan Khamin-Khao-Padtalod
Use: Folk medicine
Critical remarks:
Cultivated: MRG021, Bangkok Herbarium (BK), August 23, 2015

22. *Curcuma* 'Wan Chai-Dam'

Corm ovoid, inside bluish gray, 4 x 2.5-5 cm; **rhizome** branched, bluish gray, **Leafy shoot** 80-100 cm tall; **bladeless sheath** green or reddish, glabrous, 5-30 cm long. **Leaf-sheath** glabrous, green with red streak; **ligule** membranous, bi-lobed, 2.5 mm long; **petiole** glabrous; **blade** obovate-lanceolate, green with red patches on either sides of midrib, glabrous on both surfaces, base cuneate, apex acute, 30-50 x 7-15 cm. (no flower throughout this study)

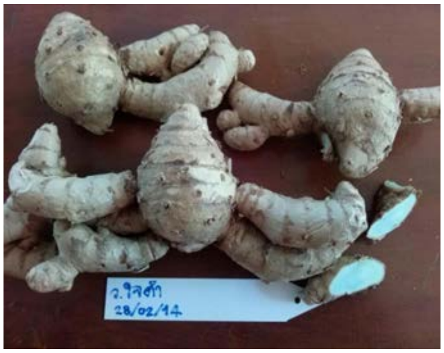

Thailand: Cultivated
Vernacular name: Wan Chai-Dam
Use: Folk medicine
Critical remarks: Rhizome color bluish gray.
Cultivated: MRG022, Bangkok Herbarium (BK), August 23, 2015

23. *Curcuma* 'Wan Khamintong'

Corm ovoid, gold yellow inside, 6-8 x 3-4 cm; **rhizome** branched, gold yellow inside. **Leafy shoot** 80-120 cm tall; **bladeless sheath** 5-30 cm long. **Leaf-sheath** glabrous; **ligule** membranous, glabrous, 3 mm long; **petiole** glabrous; **blade** lanceolate, glabrous, base cuneate, apex acuminate 60-80 x 12-16 cm. (no flower throughout this study)

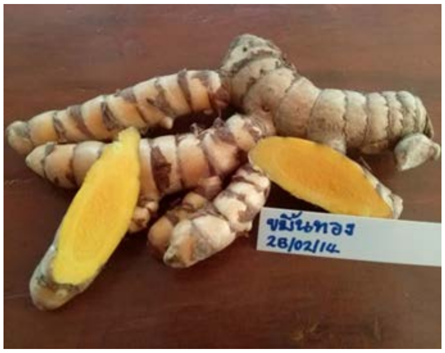

Thailand: Cultivated
Vernacular name: Wan Khamintong
Use: Spice and medicine
Critical remarks:
Cultivated: MRG023, Bangkok Herbarium (BK), August 23, 2015
